# Strong second-harmonic generation by sublattice polarization in non-uniformly strained monolayer graphene

**DOI:** 10.1038/s41467-023-38344-5

**Published:** 2023-05-04

**Authors:** Kunze Lu, Manlin Luo, Weibo Gao, Qi Jie Wang, Hao Sun, Donguk Nam

**Affiliations:** 1 School of Electrical and Electronic Engineering, Nanyang Technological University, Singapore, Singapore; 2 Division of Physics and Applied Physics, School of Physical and Mathematical Sciences, Nanyang Technological University, Singapore, Singapore; 3 Institute for Functional Intelligent Materials, National University of Singapore, Singapore, Singapore

**Keywords:** Nonlinear optics, Nonlinear optics, Optical properties and devices

## Abstract

Despite the potential of graphene for building a variety of quantum photonic devices, its centrosymmetric nature forbids the observation of second harmonic generation (SHG) for developing second-order nonlinear devices. To activate SHG in graphene, extensive research efforts have been directed towards disrupting graphene’s inversion symmetry using external stimuli like electric fields. However, these methods fail to engineer graphene’s lattice symmetry, which is the root cause of the forbidden SHG. Here, we harness strain engineering to directly manipulate graphene’s lattice arrangement and induce sublattice polarization to activate SHG. Surprisingly, the SHG signal is boosted 50-fold at low temperatures, which can be explained by resonant transitions between strain-induced pseudo-Landau levels. The second-order susceptibility of strained graphene is found to be larger than that of hexagonal boron nitride with intrinsic broken inversion symmetry. Our demonstration of strong SHG in strained graphene offers promising possibilities for developing high-efficiency nonlinear devices for integrated quantum circuits.

## Abstract (web summary)

Nanostructured platforms for efficient nonlinear optics are the building block of next generation integrated photonic devices. Here the authors provide a straightforward method to engineer the lattice symmetry of monolayer graphene, boosting the SHG signal at low temperature.

## Introduction

Nonlinear optics is a branch of modern optics with many essential applications ranging from material analysis to quantum computing^1–3^. Among many nonlinear processes, second-order *χ*^(2)^ nonlinear processes such as second-harmonic generation (SHG)^4^ and spontaneous parametric down-conversion (SPDC)^5–7^ are most frequently used in many applications owing the high *χ*^(2)^ susceptibility. The *χ*^(2)^ processes are only possible in materials with broken inversion symmetry. Some of these materials, such as beta barium borate (BBO)^8^, are extensively used in various applications as they are commercially available. However, these traditional materials have crucial limitations, including low nonlinear susceptibility and phase-matching issues^9^. Most importantly, those materials only exist in the form of bulk crystals, thus prohibiting them from being scaled down^10^. Towards the realization of miniaturized, integrated nonlinear photonic devices, the search for materials that can exist in nanometer dimensions with uncompromised nonlinear properties has become an important research topic.

Recently, atomically thin two-dimensional (2D) materials have shown great promise since many of them such as tungsten diselenide (WSe_2_)^11^ and hexagonal boron nitride (hBN)^12^ intrinsically possess broken inversion symmetry. In addition to their strong *χ*^(2)^ responses, the atomically thin nature of these 2D materials allows overcoming the phase-matching restriction, which has otherwise been a major challenge in developing nonlinear optical devices. Among all the 2D materials, graphene is the most researched material and has thus far attained the greatest amount of attention owing to its superior mechanical^13^, electrical^14^, and optical properties^15^. For example, graphene’s excellent carrier mobility and universal absorption make it an unparalleled material for many optoelectronic applications^16–19^. Over the past decade, there has been a vast amount of research works on nonlinear optical responses in graphene^17,20–23^, including saturable absorption^20^, four-wave mixing^21,22^, and third harmonic generation (THG)^23^. Unfortunately, however, graphene does not allow any intrinsic *χ*^(2)^ processes because of its preserved inversion symmetry. To break the inversion symmetry in graphene, researchers have recently devised various methods such as applying external electrical bias^24–27^, inducing in-plane optical fields^24–28^, and exploiting interlayer interaction in multilayer graphene^25,29,30^. However, all these methods do not provide any insight into breaking the lattice inversion symmetry within one layer of graphene, which is crucial in understanding how graphene’s lattice can be further engineered at the most fundamental level. Furthermore, the second-order response created by the in-plane photon momentum is considered weak since the SHG intensity is only proportional to the photon momentum^31^. Lastly, the requirements of supplying continuous electrical energy and using oblique incidence of optical pumping^27^ makes these methods unsuitable for making integrated graphene devices.

Herein, we report an experimental observation of a new type of SHG from monolayer graphene deposited on our unique strain-engineering platform. The non-uniform strain in graphene induces a strong polarization between graphene’s two sublattices that are originally balanced. This polarization breaks the sublattice symmetry in graphene, thus enabling a strong second-order response in graphene. The experimentally observed SHG in strained graphene exhibits unconventional features including a significant temperature dependence that is not previously reported graphene. In particular, we observe a nearly 50-time enhancement in graphene’s *χ*^(2)^ response when the temperature of the graphene is reduced to 4 K, which can be explained by resonantly enhanced optical transitions between pseudo-Landau levels (pLLs). Moreover, the experimental benchmarking of our strained graphene against a well-investigated monolayer hBN with an intrinsically broken inversion symmetry allows us to conclude that our strained graphene possesses a 30% larger second-order nonlinearity than hBN. As a result, our approach utilizing unique non-uniform strain and sublattice polarization presents new possibilities for fashioning the *χ*^(2)^ responses in graphene by fundamentally engineering the material’s symmetry properties. Additionally, our work provides a very convenient, stable, and scalable way to generate the second-order nonlinearity for integrated graphene devices, which holds the promise of practical optoelectronic applications. We believe that this work opens new opportunities for graphene as a promising material for the on-chip nonlinear optoelectronic devices.

## Results

### Strain engineering and sublattice polarization

**Fig. 1:**
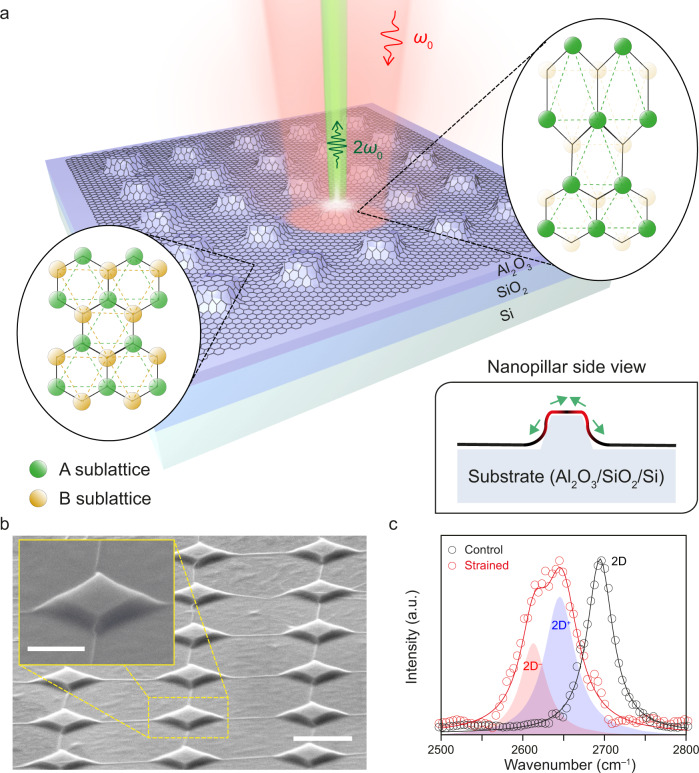
Non-uniform strain and sublattice polarization in monolayer graphene transferred on a nanopillar array. **a** Schematic illustration of strained graphene sample on a nanopillar array, which enables second harmonic generation (green light) only near the edge of the nanopillars upon the excitation of ultrafast lasers (red light). Bottom-right inset, cross-sectional schematic of monolayer graphene on a single nanopillar showing that non-uniform strain is induced near the edge of the nanopillars (red gradient color). Left eclipse, schematic of the bond structure of unstrained graphene away from the pillars showing that its sublattice preserves symmetry. Right eclipse, schematic of the bond structure of strained graphene near the edge of the pillars showing that sublattice symmetry is broken. The green and yellow circles represent the A and B sublattices, respectively. The color contrast of the circles indicates that the local density of states of the two sublattices are unbalanced in strained graphene. **b** Tilted-view SEM image of a strained graphene nanopillar array. Scale bar, 1 μm. Inset, zoom-in view of a single nanopillar. Scale bar, 500 nm. **c** Raman spectra of unstrained (black) and strained (red) graphene. In strained graphene, the Raman spectrum splits into two peaks, labeled 2D^−^ (red) and 2D^+^ (blue).

Figure 1a presents a schematic illustration of our graphene device, showing a monolayer graphene sheet conformingly adhered to the topology of the patterned nanopillar substrate. The side-view schematic in the right bottom inset to Fig. 1a highlights the induced non-uniform strain at the edges of a nanopillar in a red gradient color. The device possesses two contrasting spatial areas, an unstrained region (left ellipse) and a strongly strained region (right ellipse). The honeycomb atomic arrangement in the unstrained region presents two identical and oppositely oriented A (green dots) and B (yellow dots) sublattices^32,33^. The two sublattices possess an equal local density of states (LDOS)^33^. Therefore, the sublattice polarization becomes zero and the sublattice symmetry is preserved. In the strained region, contrastingly, a strong non-uniform strain at the edge of the nanopillar can significantly deform the carbon-carbon bonds and enables one sublattice (dark green dots) to obtain a significantly higher LDOS than the other (light yellow dots), as illustrated in the right ellipse^34,35^. This imbalance of the LDOS between the two sublattices results in a non-zero sublattice polarization that breaks the sublattice symmetry. While the existence of this sublattice polarization in graphene has previously been confirmed by scanning tunneling microscopy (STM)^34,35^, the influence of which on the optical properties of graphene has not been experimentally studied yet. Here, we exploit this sublattice polarization to observe a strong and unconventional SHG (green beam) upon the excitation of femtosecond laser pulses (red beam) (See Methods for detailed explanations for optical measurements).

Figure 1b shows the scanning electron microscopy (SEM) image of the strained graphene device used in our study. For device fabrication, we dry-transferred a monolayer graphene sheet onto an array of nanopillars to induce the sublattice polarization of graphene (See “Methods”, Supplementary Note 1 and Supplementary Fig. S1 for a detailed explanation of the fabrication procedure). The nanopillars are square-shaped with a side length of ~500 nm and a height of ~85 nm. The separation between neighboring nanopillars is 1 μm. The SEM image reveals that the exfoliated graphene flake sits on top of ~15 nanopillars, without showing any observable fracture at the sharp edges of the nanopillars.

To quantitatively obtain the amount of the induced strain in graphene, we performed Raman spectroscopy in strained graphene on the nanopillar (red, Fig. 1c) (see “Methods” for a detailed explanation of Raman spectroscopy measurements). A Raman spectrum of graphene in the unstrained region is plotted together for comparison (black, Fig. 1c). The Raman 2D peak of graphene under strain redshifts and splits into two separate peaks denoted as 2D^−^ and 2D^+^. We observe a Raman peak shift of 82.6 cm^−1^ between the 2D peak of unstrained graphene and the 2D^−^ peak of the deformed graphene, yielding a strain of ~1.26%^36^ (see “Methods” for calculation details). By considering the theoretical lattice deformation^37,38^, we calculate the maximum local atomic strain of ~2.4%, which is larger than the measured value of ~1.26%. The discrepancy is ascribed to the limited spatial resolution of the Raman spectroscopy due to a large laser spot size compared to the sharp edges of our graphene nanopillars^38,39^.

### Second harmonic generation in strained graphene

**Fig. 2:**
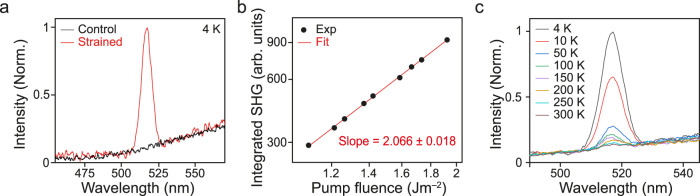
Creation of second harmonic generation in periodically strained graphene. **a** Emission spectra of pristine (black) and strained (red) graphene measured at 4 K. Both spectra show a broadband ultrafast photoluminescence, while strained graphene also shows a very sharp second harmonic generation peak at half of the pump wavelength. **b** Log plot of pump fluence dependence of integrated second harmonic generation. The slope of the plot is ~2, confirming the nature of the SHG emission. **c** Emission spectra of strained graphene at different temperatures between 4 and 300 K. As temperature decreases, the second harmonic generation response becomes more distinctive. The intensity of second harmonic generation at 4 K is ~50 times larger than that at 300 K.

We compare the emission spectrum between the control and our strained graphene devices. The control device is made with a monolayer graphene sheet exfoliated onto a plain substrate with no topology. Both the control and strained devices are in contact with a ~20-nm-thin Al_2_O_3_ layer. A customized confocal photoluminescence setup with a 1035-nm femtosecond pulsed pump laser was used (see Supplementary Note 2 for a detailed explanation of our photoluminescence setup). Figure 2a shows the emission spectra from both the control (black) and strained (red) samples measured at a cryogenic temperature of 4 K. The emission from the control sample spans across the measurement window (450 nm ~ 580 nm) without any distinct feature, which is the typical ultrafast photoluminescence spectrum of excited electrons in graphene^40,41^. Strikingly, in addition to the ultrafast photoluminescence, the strained graphene sample exhibits a very strong and sharp peak at a wavelength of 517.5 nm, which is exactly half of the pump wavelength of 1035 nm. To confirm the nature of this strong peak, we performed the pump fluence-dependent photoluminescence measurement of the integrated SHG intensity. Figure 2b shows the typical quadratic relationship between the integrated emission intensity of the SHG signal and the pump fluence, which confirms that the emission is the SHG. To confirm that the SHG originates from the strained graphene layer but not from the substrate, we performed the same measurements on the same patterned nanopillar substrate without graphene, which showed no observable SHG signal (See Supplementary Note 8 for further details on this additional measurement).

To further investigate the origin of the SHG, we performed temperature-dependent measurements. Strikingly, at a low temperature of 4 K, the observed SHG emission is almost 50 times higher than that at room temperature (Fig. 2c). The strong temperature dependence of SHG has never been reported in graphene. To briefly summarize, previous studies ascribed the observed SHG from monolayer graphene to temporarily broken inversion symmetry via in-plane optical fields^24–28^, or applied external electrical fields^24–27^, none of which has reported any temperature dependence of the SHG signal. Via current–voltage (*I*–*V*) measurements, we confirmed that the graphene layer used in our study is close to intrinsic (Supplementary Fig. S2), which excludes the possibility of the doping-induced effect for the observed SHG in the device^27^. Therefore, our experimental results suggest the necessity of introducing a new type of SHG that has yet to be discussed.

### Theoretical modeling for explaining strong and temperature-dependent SHG

**Fig. 3:**
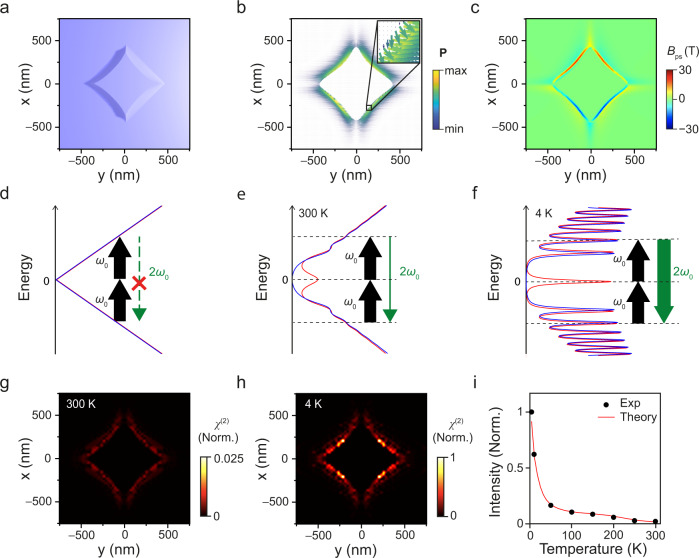
Strong temperature dependence of second harmonic generation in strained graphene. **a** Model of an isolated reconstructed nanopillar. **b**, **c** 2D mapping of sublattice polarization (**b**) and pseudo-magnetic fields (**c**) based on the reconstructed nanopillar in (**a**). **d**–**f** Illustration of density of states in unstrained graphene (**d**), strained graphene at room temperature (**e**) and strained graphene at low temperature (**f**). The red and blue curves represent the LDOS of A and B sublattices, respectively. Second harmonic generation becomes possible in strained graphene due to the sublattice polarization. At a low temperature, resonant second harmonic generation between pseudo-Landau levels can be strongly enhanced. **g**, **h** 2D mapping of second-order susceptibility in a single reconstructed nanopillar at 300 K (**g**) and at 4 K (**h**). **i** Plot of integrated second harmonic generation as a function of temperature. Black circles: experimental data; Red curve: calculated data.

To provide a quantitative explanation of our measurement results, we first reconstruct an isolated nanopillar with strained graphene (Fig. 3a), which has a side length of 500 nm and a height of 85 nm based on our atomic force microscopy (AFM) result. Using the topology of the reconstructed nanopillar model, we calculate the local strain distribution on graphene^38^. It is well reported that the local strain distribution determines the extent of sublattice polarization in graphene^42^. However, a quantitative representation of sublattice polarization is currently still lacking. On the other hand, it is also well known that the localized strain can induce artificial gauge fields or pseudo-magnetic fields (PMF) that can generate discrete pLLs^37,43,44^. Here, we develop a new theoretical framework that provides a relationship between non-uniform strain, sublattice polarization, and PMF (See Supplementary Note 3 for a detailed explanation of the theoretical framework). In this framework, we define the sublattice polarization **P** to be:

1 $${{{{{\bf{P}}}}}}=\mathop{\sum}\limits_{n}\frac{{d}_{n}}{{a}_{0}}{{{{{{\boldsymbol{\delta }}}}}}}_{n},\,(n=1,2,3)$$

where *a*_0_ (~0.14 nm) is the bond length of pristine graphene, *d*_*n*_ is the bond length along the direction **δ**_*n*_ after strain. **δ**_*n*_ refers to the three nearest neighbor vectors of a carbon atom (see Supplementary Fig. S4), where:

2 $${{{{{{\boldsymbol{\delta }}}}}}}_{1}={a}_{0}\left(\frac{\sqrt{3}}{2},\frac{1}{2}\right),\,{{{{{{\boldsymbol{\delta }}}}}}}_{2}={a}_{0}\left(-\frac{\sqrt{3}}{2},\frac{1}{2}\right),\,{{{{{{\boldsymbol{\delta }}}}}}}_{3}={a}_{0}\left(0,-1\right).$$

Additionally, we derive a direct relationship between **P** and PMF (see Supplementary Note 3 for a detailed mathematical derivation):

3 $${B}_{{{{{{\rm{ps}}}}}}}=\frac{2\beta }{3{a}_{0}^{2}}{{{{{\boldsymbol{\nabla }}}}}}\cdot {{{{{\bf{P}}}}}}$$

where *β* (~3) is a constant and **∇** · **P** is the divergence of **P**. Figure 3b, c displays the calculated 2D mappings of **P** and *B*_ps_, respectively. **P** (Fig. 3b) reaches its maximum values near the sharp edges of the nanopillars, where the graphene experiences the largest deformation, while PMF (Fig. 3c) attains a maximum value of ~30 T where the strain gradient is the steepest^38,45^.

The PMF generated at the edges of the nanopillar creates discrete pLLs in graphene, which can strongly modify the electrical and optical properties of graphene^38,46,47^. Previously, the creation of Landau levels by external magnetic fields in gallium arsenide (GaAs)^48^ and graphene-based heterostructures^31^ was theoretically predicted to activate a strong and resonant SHG when the SHG energy matches with inter-Landau level separation. Here, we theoretically extend this phenomenon of the resonant SHG to the case of strained graphene possessing a strong PMF. We show that the resonant SHG via inter pLLs in our strained graphene sample has a strong temperature dependence. First, by using rigorous tight-binding simulations, we calculate the distribution of density of states (DOS) in pristine and strained graphene (see Supplementary Note 4 for tight-binding simulations). Figure 3d–f show schematic illustrations of the DOS for pristine graphene, strained graphene at 300 K, and strained graphene at 4 K, respectively. The red and blue curves represent the LDOS of A and B sublattices, respectively. The preserved inversion symmetry in pristine graphene, which is highlighted by the overlapping LDOS curves, prevents the occurrence of the SHG process (Fig. 3d). In the strained graphene sample under non-uniform strain (Fig. 3e), a significant sublattice polarization can be induced at the edges of nanopillars as shown in Fig. 3b, which breaks the inversion symmetry in graphene and leads to the splitting of LDOS between the A and B sublattices (See Supplementary Note 4 for detailed calculation on LDOS and Supplementary Fig. S4 for the distribution of LDOS). Additionally, the strain-induced PMF creates the pLL peaks^35^, which are discrete energy levels with high DOS (Fig. 3e)^37^. In this strained graphene system, the SHG can be enabled (highlighted by a downward green arrow) because of the sublattice polarization-induced *χ*^(2)^ nonlinearity. Also, the magnitude of SHG can be strong owing to the resonant optical transitions between pLLs. It should be noted that in pseudo-Landau-quantized graphene, the resonant SHG can be considered as two successive one-photon transitions. Each of these transitions must obey the optical selection rule of ${{{{{{\rm{|}}}}}}l}_{f}{{{{{\rm{|}}}}}}-{{{{{{\rm{|}}}}}}l}_{i}{{{{{\rm{|}}}}}}=\pm 1$, where *l*_*i*_ and *l*_*f*_ are the pLL index of the initial and final states respectively. As shown in Fig. 3f, the SHG can be further enhanced at low temperatures (highlighted by a thick downward green arrow) because the reduced thermal broadening of pLLs leads to sharper, higher-density electronic states at a certain energy level, further increasing resonant optical transitions between pLLs.

We also calculate the spatial distribution of the *χ*^(2)^ strength in strained graphene based on a well-reported method of nonlinear bubble diagrams^31^ (see Supplementary Note 5 for the detailed calculation procedure). Figure 3g, h shows the 2D mappings of normalized *χ*^(2)^ at 300 K and 4 K, respectively, assuming that the excitation laser wavelength is 1035 nm. At 300 K, local *χ*^(2)^ at the edges of the nanopillar attains a considerably high value compared to areas away from the nanopillar, owing to the strong sublattice polarization and the resonant SHG enabled by pLLs at these locations. The distribution of *χ*^(2)^ becomes more interesting at 4 K, where *χ*^(2)^ becomes extremely large at only certain locations along the edges of the nanopillar (Fig. 3h). In these bright spots, the induced PMF can obtain specific magnitudes, at which the resonant optical transition between pLLs can enhance the *χ*^(2)^ value significantly only at low temperatures (see Supplementary Note 5 and Supplementary Fig. S5 for the effect of the magnitudes of PMF on *χ*^(2)^). Meanwhile, we also consider the temperature-dependent phonon-assisted scattering events which mediate the efficiency of the SHG emission. When the temperature decreases, the scattering rate between the carriers and phonons decreases, leading to a more dominant SHG process, therefore favoring a stronger SHG emission (see Supplementary Note 6 for quantitative calculations of the effect of acoustic and optical phonons).

Lastly, by adding up the *χ*^(2)^ value over an entire area shown in Fig. 3g, h, we can obtain the relative SHG intensities at different temperatures. Figure 3i shows the calculated (red curve) and experimental (black dots) intensity of SHG at various temperatures. Our calculated results reveal a significant enhancement of the SHG intensity at lower temperatures, which is in excellent agreement with our experimental results. It is worth mentioning that the resonant condition for SHG in our strained graphene is expected to be consistently met. This is due to the energetically dense distribution of pLLs compared to the laser photon energy and the spatial variability of pLLs across the nanopillar, which provides various possible pLLs transition combinations for resonant SHG. In contrast, if resonant transitions for SHG are not considered, the SHG intensity changes only modestly with temperature, failing to explain the remarkable temperature-dependent behavior observed in our experiments (see Supplementary Fig. S17).

## Discussion

In summary, we have demonstrated the experimental observation of SHG from strained graphene on nanopillar structure. The SHG arises from the sublattice symmetry breaking in graphene due to the non-uniform strain induced by the nanopillars. At a low temperature of 4 K, the SHG intensity is almost 50 times higher than that at room temperature. The drastic enhancement of SHG intensity is explained by the inter-pLL resonance when the strain-induced PMF is present. At a low temperature, the DOS at each pLL is significantly increased owing to a reduced thermal broadening, which strongly enhances the resonant inter-pLL optical transitions and SHG when the energy separation between pLLs matches the SHG energy.

Lastly, we estimate the conversion efficiency of the SHG and the sheet second-order susceptibility ${\chi }_{{{{{{\rm{sh}}}}}}}^{(2)}$ of the strained graphene^49^ (See Supplementary Note 9 for details on this estimation). By comparing the emitted SHG intensity against the laser excitation intensity, we estimate the conversion efficiency to be in the order of 10^−9^ and the ${\chi }_{{{{{{\rm{sh}}}}}}}^{(2)}$ is calculated to be 10^−20^ m^2^ V^−1^. Meanwhile, we also derive a similar value of ${\chi }_{{{{{{\rm{sh}}}}}}}^{(2)}$ by benchmarking the emission spectrum of the strained graphene against that of hBN whose inversion symmetry is intrinsically broken (Supplementary Fig. S7). Strikingly, the ${\chi }_{{{{{{\rm{sh}}}}}}}^{(2)}$ value of our strained graphene is 30% larger than that of hBN, which proves that our strain-engineered graphene is a new, promising material for 2D materials-based second-order nonlinear photonics.

One interesting observation in our experiment is that the emitted SHG is highly anisotropic and shows twofold symmetric polarization patterns when graphene is strained in one direction on a trench (see Note 10 of Supplementary Information). This shows that the strain-induced sublattice polarization can be used to modify the point symmetry of graphene and allows us to design new anisotropic SHG patterns. Additionally, we also observe that the intensity of SHG increases when the average height of the nanopillar array increases (see Note 11 of Supplementary Information). This can be explained with the fact that a large PMF forces the LDOS of pseudo-Landau levels to be sharper, and therefore enables a stronger resonance between the pseudo-Landau levels and produces a large SHG intensity. Lastly, we also observe that the SHG intensity in our strained graphene can be tuned by the back gate voltage (see Note 12 of Supplementary Information). Due to the effect of Pauli blocking^50^, the SHG intensity reduces drastically when the back gate voltage is tuned away from the Dirac point voltage.

One important implication of our study on SHG is the realization of graphene-based SPDC, which is a reversed process of SHG that involves the emission of two entangled photons with exactly the same properties. SPDC based on 2D materials has been attracting increasing interest recently as it is a crucial process for the realization of quantum circuits based on 2D materials^51–53^. The study of strain-induced nonlinear characteristics in graphene can also be further extended to third harmonic generation or other nonlinear processes, which can be of great use in other nonlinear optoelectronic devices. In addition, circular polarization-dependent or valley-dependent nonlinear processes could be possible in pseudo-Landau-quantized graphene system^54,55^, which adds another dimension to the study of nonlinear processes. Interestingly, our simulation result reveals that the sublattice polarization can significantly suppress the LDOS at the zeroth pseudo-Landau level of one sublattice while that of another sublattice remains very large (Supplementary Fig. S4d), potentially enabling a new degree of freedom for pseudo-spin using strain-induced sublattice polarization^56^. It is also noteworthy that such LDOS polarization can be tunable via applying a dynamic strain tuning method^57^. We believe that our strained graphene platform provides promising possibilities for investigating a rich class of nonlinear optical phenomena, which has huge potential in integrated quantum photonics applications.

## Methods

### Device fabrication

#### Nanostructured array

The nanostructured substrate was patterned using e-beam lithography (EBL) and a wet chemical approach. Patterns for the square arrays were firstly defined in a 380 nm spin-coated polymethyl methacrylate (PMMA) layer. Subsequently, using the patterned PMMA as an etch mask, the SiO_2_ layer was etched in buffered oxide etch (BOE) (12.5% HF, 87.5% NH4F) for 3 min 30 s, forming an array of nanopillars. The square pillars have 500-nm side length and 85-nm height. The whole chip was coated with a 20-nm Al_2_O_3_ layer by atomic layer deposition before graphene dry transfer.

#### Graphene exfoliation and transfer

Graphene flakes were obtained with Scotch tape by mechanical exfoliation of a small piece of Kish graphite (Graphene Supermarket). The tape with thinned graphene flakes was pushed down onto a polydimethylsiloxane (PDMS) film and then detached. The monolayer flake on PDMS was found under an optical microscope, and then the number of layers was confirmed using Raman spectroscopy. The graphene flake was then aligned and stamped on a nanostructured substrate with an all-dry transfer method using our home-built dry transfer setup.

### Structural characterization

#### Scanning electron microscopy

The structural characteristics and morphology of the fabricated sample devices were characterized by field-emission scanning electron microscopy (SEM) (Apreo S FESEM). The electron acceleration voltage in SEM was 5 kV.

#### Raman spectroscopy and strain estimation

The thickness of graphene flakes and strain distribution in graphene on nanopillars were characterized using a WITec Raman setup with 100× objective lens (NA = 0.9) and 600 lines/mm grating. We used a 532-nm laser at a low laser power of <1 mW to avoid any heating effect. We confirm the graphene sample to be monolayer based on the observation that the 2D peak intensity is ~2 times the G peak intensity^58^. The maximum measured strain is estimated to be 1.26% by assuming a strain-shift coefficient of 65.4% cm^–1^/%^36^ between the 2D peak of unstrained graphene and the 2D^–^ peak of the strained graphene.

### Second harmonic generation measurements

#### Temperature-dependent measurement

The measurement on graphene’s SHG was performed in the reflection geometry. The sample was placed in an open-cycle cryostat (Janis ST-500) cooled by liquid helium. The temperature for our measurements varies between 4 K and 300 K. The excitation pump pulses were generated from a 1035-nm femtosecond laser with a 120-fs pulse duration and a repetition rate of 76 MHz (Flint, Light Conversion). The laser was defocused using a focal lens to cover a spot-size area of approximately 15 × 15 μm^2^ on the sample. Supplementary Fig. S3 provides a detailed schematic illustration of the temperature-dependent measurement setup.

## Supplementary information

Supplementary Information

## Data Availability

The data that support the findings of this study are available within the main text and Supplementary Information. Any other relevant data are available from the corresponding authors upon reasonable request.
