# Supplementary material for: Strong second-harmonic generation by sublattice polarization in non-uniformly strained monolayer graphene
Source: Nat Commun. 2023 May 4;14:2580. doi: 10.1038/s41467-023-38344-5 (PMC10160016; doi:10.1038/s41467-023-38344-5)
Supplement: Supplementary file 1 — Supplementary Information [file 41467_2023_38344_MOESM1_ESM.pdf]

Supplementary Information

Strong second-harmonic generation by sublattice polarization in non-uniformly strained monolayer graphene

---

Table of Content

Note 1. Fabrication procedure and characterization

Note 2. Measurement setup

Note 3. Theoretical calculation of lattice polarization and pseudo-magnetic fields

Note 4. Theoretical calculation of density of states

Note 5. Theoretical calculation of second-order susceptibility

Note 6. Decay of excited carrier

Note 7. General expression of the strain-induced gauge field

Note 8. Background SHG

Note 9. Estimation of conversion efficiency and second-order susceptibility

Note 10 Polarization-resolved SHG

Note 11 SHG from graphene on pillar arrays with different heights

Note 12 SHG from strained graphene under gating

Supplementary Figures S1–S17

References

Note 1. Fabrication procedure and characterization

Fabrication of nanostructured array. Supplementary Fig. S1 provides the detailed schematic illustration for fabricating the nanostructure array. We first spin-coated a polymethyl methacrylate (PMMA) resist (950 PMMA A6, MICROCHEM) on a 300-nm-thick SiO_2/Si substrate at 4500 rpm for 30 sec, followed by baking at 180 ºC for 2 min (Supplementary Figs. S1a-b). Then, an etching mask was patterned (Supplementary Fig. S1c) using Raith e-line e-beam lithography system. The substrate was then immersed in buffered oxide etch (BOE) (12.5% HF, 87.5% NH_4F) with 3 min 30 sec at room temperature (Supplementary Fig. S1d). PMMA resist was removed by acetone, isopropyl alcohol (IPA), deionized (DI) water, and O_2 plasma treatment (150 W, 3 min), followed by atomic layer deposition (ALD) to deposit a 20-nm Al_2O_3 layer on the entire substrate (Supplementary Figs. S1e-f).

Dry transfer of graphene. Graphene flakes were obtained with Scotch tape by mechanical exfoliation of a small piece of Kish graphite (GRAPHENE SUPERMARKET). The tape with thinned graphene flakes was pushed down onto a polydimethylsiloxane (PDMS) film and then detached. The monolayer flake on PDMS was found under an optical microscope, and then the thickness was confirmed using Raman spectroscopy. The graphene flake was then inverted, aligned, and stamped on a nanostructured substrate with an all-dry transfer method using our home-built dry transfer setup. The temperature was maintained at 90 °C for 15 minutes before PDMS is detached.

I-V measurement of graphene. Graphene transferred onto the nanopillar array may be prone to substrate-induced charges, which could complicate subsequent measurements on the SHG. Therefore, it is crucial to confirm that the charges induced by the underlying Al_2O_3 in graphene is negligible by measuring its I_d-V_g characteristics. Supplementary Fig. S2 shows the I-V characteristics of the graphene on Al_2O_3 substrate. The Dirac point is close to 0, which means that the graphene can be regarded as undoped. Therefore, the effect of doping on SHG is neglected in our study.

Note 2. Measurement setup

Optical measurements on SHG are performed using our home-built free-space measurement setup, as illustrated in Supplementary Figure S3. The fabricated samples are placed in an open-cycle cryostat (Janis ST-500). The cryostat is then vacuumed to less than 1×10^{-6} Torr before liquid helium is used to lower the temperature. The cryostat has a built-in temperature controller, allowing us to adjust temperature between 4 K to 300 K.

As shown in Supplementary Figure S3, the graphene sample is excited by a linearly polarized 1035 nm femtosecond laser with a repetition rate of 76 MHz and a pulse duration of about 120 fs (Flint). The laser is focused by a $50 \times$ objective lens with a numerical aperture of 0.42 and shines normally onto the sample. The emitted SHG from graphene is collected using the reflection geometry by the same objective lens, and the signal is directed to the spectrometer (Princeton Instrument) and detector (Andor CCD). During the SHG measurements, the grating of the spectrometer is set to 600 grooves per millimeter. The emission spectrum can be directly captured by the detector after background subtraction. A typical integration time of 1 minute is used in our measurement to obtain clear ultrafast photoluminescence and distinct SHG peak of graphene. To enhance the SHG signal, the pump laser is defocused using a focal lens with a reasonably long focal length. The defocused laser allows shining on a larger area of the sample, thereby enhancing the intensity of the measured signal.

Note 3. Theoretical calculation of lattice polarization and pseudo-magnetic fields

Without losing generality, we start from the tight-binding Hamiltonian with nearest-neighbor hopping (NNH) parameters on a honeycomb lattice, which is written as[1]:

$$H = \sum_{i,n} t_{\boldsymbol{r}_i,n}\, a_{\boldsymbol{r}_i}^{\dagger} b_{\boldsymbol{r}_i+\boldsymbol{\delta}_n} + h.c., \tag{3-1}$$

where $t_{\boldsymbol{r}_i,n}$ is the NNH parameter, $a_{\boldsymbol{r}_i}^{\dagger}(a_{\boldsymbol{r}_i})$ and $b_{\boldsymbol{r}_i}^{\dagger}(b_{\boldsymbol{r}_i})$ are the creation (annihilation) operators of sublattice A and B, and i runs over the positions of all unit cells. The

pristine graphene geometry can be described by three nearest neighbor vectors as shown in Supplementary Fig. S4a:

$$\boldsymbol{\delta}_1 = \mathrm{a}_0\left(\frac{\sqrt{3}}{2},\frac{1}{2}\right), \boldsymbol{\delta}_2 = a_0\left(-\frac{\sqrt{3}}{2},\frac{1}{2}\right), \boldsymbol{\delta}_3 = a_0(0,-1), \tag{3-2}$$

The lattice deformation effect can be incorporated into the NNH parameters $t_{\boldsymbol{r}_i,n}$, and its explicit form in exponential approximations can be written as:

$$t_{r_i,n} = t_0 e^{-\beta\left(\frac{d_{i,n}}{a_0}-1\right)} = t_0 + \delta t_n(\mathbf{r}_i), \tag{3-3}$$

where $t_0 = 2.7\, eV$ and $a_0 = 0.14\, nm$ are the NNH parameter and bond length, respectively. $\beta = |\partial \log t / \partial \log a_0| \approx 3$. $\delta t_n(\boldsymbol{r_i})$ varies in space and is dominated by the deformation.

We show the modulation term $\delta t_n(\mathbf{r}_i) = t_0\left(e^{-\beta\left(\frac{d_{i,n}}{a_0}-1\right)} - 1\right)$ gives rise to the gauge field.

By employing Fourier transformations of Eq. (3-1) to wave vector space, and linearly expanding the whole Hamiltonian near the one of the Dirac points: $\mathbf{K} = \left(\frac{4\pi}{3\sqrt{3}a_0}, 0\right)$, we obtain the low energy effective model:

$$H = v_0\boldsymbol{\sigma}\cdot\mathbf{q} + v_0\boldsymbol{\sigma}\cdot\mathbf{A} + O_h(\delta t_n q), \tag{3-4}$$

where $v_0 = \frac{3a_0t_0}{2}$ is the Fermi velocity. The first term $v_0\boldsymbol{\sigma}\cdot\boldsymbol{q}$ is typical Dirac Hamiltonian of pristine graphene. The second term contains the general expressions of strain-induced gauge field. Explicitly, we have:

$$A_x = \frac{1}{3a_0t_0}(\delta t_1 + \delta t_2 - 2\delta t_3), A_y = \frac{\sqrt{3}}{3a_0t_0}(\delta t_2 - \delta t_1). \tag{3-5}$$

The last term in Eq. (3-4) will give rise to the modulation of Fermi velocity and an additional high-order geometric gauge field, which will be discussed later. Alternatively, the gauge field can be also written in a more compact form:

$$\mathbf{A} = \frac{1}{v_0}\sum_n \delta t_n \mathbf{T}_{K,n}, \tag{3-6}$$

where $\mathbf{T}_{K,n} = (\cos(\mathbf{K}\cdot\boldsymbol{\delta}_n), \sin(\mathbf{K}\cdot\boldsymbol{\delta}_n))$ is the geometric vector of graphene, which has the relation with nearest neighbor vector $\boldsymbol{\delta}_n = a_0\left(T_{K,n}^y, -T_{K,n}^x\right)$. Since vector potential is gauge variant, it can also be written as the function of hopping parameters:

$$\mathbf{A} = \frac{1}{v_0}\sum_n t_n \mathbf{T}_{K,n}. \tag{3-7}$$

The gauge field is valley-dependent and has opposite signs in two valleys due to the protection of time-reversal symmetry. The PMF is the curl of the gauge field and can be written as:

$$B_p = \nabla \times \mathbf{A} = \epsilon^{ab}\,\partial_a A_b = \frac{1}{v_0}\sum_n \left(T_{K,n}^{y}\,\partial_x \delta t_n - T_{K,n}^{x}\,\partial_y \delta t_n\right), \tag{3-8}$$

where ϵ^{ab} is the antisymmetric tensor. We can define a conjugate vector of strain-induced gauge field $\boldsymbol{A}$, which has the form:

$$\mathbf{F} = \frac{-2}{3a_0 t_0}\left(\sum_n T_{K,n}^{y}\delta t_n, -\sum_n T_{K,n}^{x}\,\delta t_n\right) = \frac{-2}{3a_0 t_0}\sum_n \delta\mathbf{t}_n, \tag{3-9}$$

and maintains $\mathbf{F}\cdot\mathbf{A} = 0$, where $\delta\mathbf{t}_n = \frac{\boldsymbol{\delta}_n}{a_0}\delta t_n$, and PMF B_p can be rewritten as:

$$B_p = \nabla\cdot\mathbf{F} \tag{3-10}$$

Since $\delta t_n = t_0\left(e^{-\beta\left(\frac{d_n}{a_0}-1\right)} - 1\right) \approx t_0\beta - t_0\beta\frac{d_n}{a_0}$, we can obtain:

$$\mathbf{F} \approx \frac{-2}{3a_0 t_0}\sum_n \frac{\boldsymbol{\delta}_n}{a_0}\left(t_0\beta - t_0\beta\frac{d_n}{a_0}\right) = \frac{2\beta}{3a_0^2}\sum_n \frac{d_n}{a_0}\boldsymbol{\delta}_n = \frac{2\beta}{3a_0^2}\mathbf{P}. \tag{3-11}$$

where $\mathbf{P} = \sum_n \frac{d_n}{a_0}\boldsymbol{\delta}_n$ is the lattice polarization vector.

Note 4. Theoretical calculation on the density of states in strained graphene

We can also directly calculate the local density of state to verify the strain-induced inversion symmetry breaking. The expression is given by:

$$D_A(\epsilon,\mathbf{r}) = \frac{1}{\pi}\sum_n \frac{\gamma}{(\epsilon-\epsilon_n)^2+\gamma^2}|c_{n,A}(\mathbf{r})|^2$$

$$D_B(\epsilon,\mathbf{r}) = \frac{1}{\pi}\sum_n \frac{\gamma}{(\epsilon-\epsilon_n)^2+\gamma^2}|c_{n,B}(\mathbf{r})|^2 \tag{4-1}$$

where $|c_{n,A}(\mathbf{r})|^2$ and $|c_{n,B}(\mathbf{r})|^2$ are the contribution weights of A and B sublattice in the LDOS at $\boldsymbol{r}$ point.

As shown in Supplementary Fig. S4b-d, the LDOS in the unstrained area away from the nanopillar (area (1) in Supplementary Fig. S4b) shows the two-fold degeneracy of the sublattice, while at the edge of the nanopillar where graphene experiences considerable strain (area (2) in Supplementary Fig. S4b), one can see the pronounced pseudo-Landau level peaks and the states splitting, which is due to the strain-induced symmetry breaking.

Note 5. Theoretical calculation of second-order susceptibility

The second harmonic generation is a nonlinear response, which can be studied using the perturbation theory. Generally, the total Hamiltonian with the perturbation is: $H = H_0 + H'$, where $H' = \sum_{nl} \frac{i\hbar e}{m_0} \mathbf{M}_{nl} \cdot \mathbf{A} a_n^\dagger a_l$ is interaction Hamiltonian between the external pump light and electrons, where $\mathbf{M}_{nl} = \langle n|\boldsymbol{\nabla}|l\rangle$ is the optical matrix element and A is the external vector potential of the pump light. The expectation value of any observable is given by $\langle O\rangle = Tr(O\rho)$. The second harmonic generation can be represented by a second-order current response, which is given by[2,3]:

$$\langle \mathbf{J}\rangle^{(2)} = Tr\big(\mathbf{J}\rho^{(2)}\big) = \sum_{m,n} \mathbf{J}_{mn}\, \rho_{nm}^{(2)} \tag{5-1}$$

where $\mathbf{J} = e\mathbf{v}$ is the current operator, v is the velocity operator, $\mathbf{J}_{mn} = \langle m|\mathbf{J}|n\rangle$ is the current matrix element and $\rho_{nm}^{(2)} = \langle n|\rho^{(2)}|m\rangle$ is the second-order density matrix element. With the perturbation H', the density matrix ρ will follow the equation of motion:

$$\frac{\partial\rho}{\partial t} = \frac{1}{i\hbar}[H,\rho] + \frac{\partial\rho}{\partial t}\Big|_{scatter} \tag{5-2}$$

where we employ the relaxation time approximation $\frac{\partial\rho}{\partial t}|_{scatter} \approx \frac{\rho_0-\rho}{\tau}$. The recursive expansion of density matrix is given by:

$$\rho^{(n+1)}(t) = \frac{i}{\hbar}\int_0^t dt' e^{-\frac{t'}{\tau}} e^{-i\frac{H_0}{\hbar}t'} \big[H'(t-t'),\rho^{(n)}(t-t')\big] e^{i\frac{H_0}{\hbar}t'} \tag{5-3}$$

Using the above recursion formula, we can quickly obtain the second-order density matrix element $\rho_{nm}^{(2)}$, which is given by:

$$\rho_{nm}^{(2)}(t) = \int \frac{d\omega d\omega'}{(2\pi)^2} e^{i(\omega+\omega')t} \frac{-1}{\epsilon_{mn} + i\hbar/\tau - \hbar(\omega+\omega')}$$

$$\sum_l \left(\frac{f_{lm} H'_{nl}(\omega') H'_{lm}(\omega)}{\epsilon_{ml} - \hbar\omega + i\hbar/\tau} - \frac{f_{nl} H'_{nl}(\omega) H'_{lm}(\omega')}{\epsilon_{ln} - \hbar\omega + i\hbar/\tau} \right) \tag{5-4}$$

where $f_{lm} = f_l - f_m$, and f_l is the Fermi distribution of the carriers at l quantum state. $\epsilon_{mn} = \epsilon_m - \epsilon_n$ is the energy difference between two quantum states. Thus, the second-order current response is given by:

$$\langle \mathbf{J} \rangle^{(2)} = \sum_{mnl} \frac{-\mathbf{J}_{mn}}{\epsilon_{mn} + i\hbar/\tau - 2\hbar\omega} \left(\frac{f_{lm} H'_{nl}(\omega) H'_{lm}(\omega)}{\epsilon_{ml} - \hbar\omega + i\hbar/\tau} - \frac{f_{nl} H'_{nl}(\omega) H'_{lm}(\omega)}{\epsilon_{ln} - \hbar\omega + i\hbar/\tau} \right)$$

$$= \frac{-\hbar^2 e^3}{\omega^2 m_0^2} \sum_{mnl} \frac{\mathbf{v}_{mn}}{\omega_{mn} - 2\omega + i\hbar/\tau} \left(\frac{f_{lm} \mathbf{M}_{nl} \mathbf{M}_{lm}}{\omega_{ml} - \omega + i\hbar//\tau} - \frac{f_{nl} \mathbf{M}_{nl} \boldsymbol{M}_{lm}}{\omega_{ln} - \omega + i\hbar/\tau} \right) : \mathbf{E}(\omega)\mathbf{E}(\omega).$$

(5-5)

where the perturbation matrix element $H'_{nl}(\omega) = \frac{i\hbar e}{m_0} \mathbf{M}_{nl} \cdot \mathbf{A}(\omega) = \frac{\hbar e}{\omega m_0} \mathbf{M}_{nl} \cdot \mathbf{E}(\omega)$, $\mathbf{v}_{mn}$ is the velocity matrix element, and : is the double-dot product symbol between the dyadic tensors $\mathbf{M}_{nl}\mathbf{M}_{lm}$ and $\mathbf{E}(\omega)\mathbf{E}(\omega)$. Thus, we have the element of nonlinear conductivity tensor of second harmonic generation is given by:

$$\sigma_{bc}^{a} = \frac{-\hbar^2 e^3}{\omega^2 m_0^2} \sum_{mnl} \frac{v_{mn}^{a}}{\omega_{mn} - 2\omega + i\hbar/\tau} \left(\frac{f_{lm} M_{nl}^{b} M_{lm}^{c}}{\omega_{ml} - \omega + i\hbar//\tau} - \frac{f_{nl} M_{nl}^{b} M_{lm}^{c}}{\omega_{ln} - \omega + i\hbar/\tau} \right). \tag{5-6}$$

This is the general expression for the second-order response. There is another equivalent method with added efficiency for the second harmonic generation in strained graphene under PMF. The method is calculated from the nonlinear bubble diagrams and is given by[4]:

$$\sigma_{bc}^{a} = 4i\omega n \sum_{n=1,s=\pm 1}^{+\infty} \Theta_{bc}^{a}(n, -n-s)[f(\epsilon_n - \mu) - f(-\epsilon_{n+s} - \mu)]$$

$$\left[\frac{1}{(2i\omega)^2 - (\epsilon_n + \epsilon_{n+s})^2} + \frac{1}{(i\omega)^2 - (\epsilon_n + \epsilon_{n+s})^2} \right], \tag{5-7}$$

where ϵ_n is the energy of the landau level, μ is the chemical potential, and $\Theta^{a}_{bc}(n, -n-s)$ is the matrix element that contains the selection rule. For more information on the calculation, one can check the Ref[4]. Supplementary Fig. S5 shows the calculated SHG intensity for a range of different magnitudes of PMF between 0 and 40 T. We observe that the intensity of SHG becomes high at certain PMF magnitudes that can enable resonant SHG. The resonance is amplified to give a sharper SHG response when the temperature of graphene is reduced.

Note 6. Decay of excited carrier

Excited carriers in graphene under ultrafast pulses will decay, and this process can be described by the formula[5]:

$$\frac{d\rho_i}{dt} = S_i^{in}(1-\rho_i) - S_i^{out}\rho_i$$
$$= \left(\Gamma_{i,c}^{in} + \Gamma_{i,p}^{in}\right)(1-\rho_i) - \left(\Gamma_{i,c}^{out} + \Gamma_{i,p}^{out}\right)\rho_i, \quad (6\text{-}1)$$

where $\Gamma_{i,c}^{in/out}$ is the Coulomb scattering rate between the carriers, and $\Gamma_{i,p}^{in/out} \propto L_\gamma(\Delta E^{em})\left(n_{ph}+1\right) + L_\gamma(\Delta E^{ab})n_{ph}$ is the scattering rate by phonons, where the phonon density n_{ph} is thermally excited, thus temperature-dependent.

In our experiments, the measured temperature-dependent SHG intensity has two distinct regimes, including a fast decay of SHG intensity at low temperatures, and a slower decay of SHG intensity when the temperature is high. This indicates two different mechanisms responsible for the thermal quenching by the equation[6]:

$$I_e = \frac{I_0}{1 + ae^{\frac{-e_1}{k_B T}} + be^{\frac{-e_2}{k_B T}}}, \quad (6\text{-}2)$$

where a and b are the coupling strength between electrons and phonon modes, and $e_1 = 6$ meV and $e_2 = 150$ meV are the activation energies corresponding to the acoustic and optical phonon processes in the carrier decay[7,8]. Based on this, we consider the acoustic ΓA and optical phonon KT modes in the decay process[5]. The acoustic phonon assists the carrier decay on the quasi-continuous high-energy pLLs. The impact of the optical phonons on the photoluminescent dynamics is determined by resonances between inter-pLL transitions.

Note 7 General expression of the strain-induced gauge field

This section gives a detailed derivation of the artificial gauge field in strained graphene. We start from the Hamiltonian in Eq. (3-1):

$$H = \sum_{i,n} t_{\boldsymbol{r}_i,n}\, a^{\dagger}_{\boldsymbol{r}_i} b_{\boldsymbol{r}_i+\delta_n} + h.c., \tag{7-1}$$

where $a^{\dagger}_{\boldsymbol{r}_i}(a_{\boldsymbol{r}_i})$ and $b^{\dagger}_{\boldsymbol{r}_i}(b_{\boldsymbol{r}_i})$ are the creation (annihilation) operators of sublattice A and B, which follow the anti-commutation relations:

$$\{a_i, a_j^{\dagger}\} = 1, \qquad \{b_i, b_j^{\dagger}\} = 1, \tag{7-2}$$

with Fourier transformation, one can have the Hamiltonian in Eq. (7-1) in k-space:

$$H(k) = \sum_n t_{\boldsymbol{r}_i,n} \begin{pmatrix} 0 & e^{-i\boldsymbol{k}\cdot\boldsymbol{\delta}_n} \\ e^{i\boldsymbol{k}\cdot\boldsymbol{\delta}_n} & 0 \end{pmatrix}. \tag{7-3}$$

We expand the matrix to the first-order of small q around the Dirac point $\mathbf{K} = \left(\frac{4\pi}{3\sqrt{3}a_0}, 0\right)$ and rewrite the hopping parameter into two parts $t_{r_i,n} = t_0 + \delta t_n(r_i)$.

$$H(q) = \sum_n (t_0 + \delta t_n(\boldsymbol{r_i})) \begin{pmatrix} 0 & e^{-i(\boldsymbol{q}+\boldsymbol{K})\cdot\boldsymbol{\delta}_n} \\ e^{i(\boldsymbol{q}+\boldsymbol{K})\cdot\boldsymbol{\delta}_n} & 0 \end{pmatrix}. \tag{7-4}$$

The constant matrix in the above expression can be simplified, which gives us:

$$\begin{pmatrix} 0 & e^{-i\boldsymbol{K}\cdot\boldsymbol{\delta}_n} \\ e^{i\boldsymbol{K}\cdot\boldsymbol{\delta}_n} & 0 \end{pmatrix} = \boldsymbol{\sigma}\cdot\mathbf{T}_{K,n}, \tag{7-5}$$

where $\mathbf{T}_{K,n} = (\cos(\mathbf{K}\cdot\boldsymbol{\delta}_n), \sin(\mathbf{K}\cdot\boldsymbol{\delta}_n))$ is the geometric vector of graphene, the Eq. (7-4) becomes:

$$\begin{aligned} H(q) &= \sum_n (t_0 + \delta t_n(\mathbf{r}_i)) \begin{pmatrix} 0 & e^{-i(\mathbf{q}+\mathbf{K})\cdot\boldsymbol{\delta}_n} \\ e^{i(\mathbf{q}+\mathbf{K})\cdot\boldsymbol{\delta}_n} & 0 \end{pmatrix} \\ &= \sum_n (t_0 + \delta t_n(\mathbf{r}_i)\boldsymbol{\sigma}\cdot\mathbf{T}_{K,n}(1 + i\sigma_3\mathbf{q}\cdot\boldsymbol{\delta}_n) \\ &\approx H_0 + H_1. \end{aligned} \tag{7-6}$$

The term H_0 is the Dirac Hamiltonian of the pristine graphene, which is given by:

$$H_0(q) = \sum_n t_0\,\boldsymbol{\sigma}\cdot\mathbf{T}_{K,n} i\sigma_3\mathbf{q}\cdot\boldsymbol{\delta}_n = v_0\sigma_a G^{ab} q_b = v_0\boldsymbol{\sigma}\cdot\mathbf{q}, \tag{7-7}$$

where $G^{ab} = -i\sigma_3 g^{ab}$, and $g^{ab} = \frac{2}{3a_0}\sum_n T^a_{K,n}\delta^b_n$, one can easily find out:

$$\sum_n \mathbf{T}_{K,n}\boldsymbol{\delta}_n = \frac{3a_0}{2}\begin{pmatrix} 0 & -1 \\ 1 & 0 \end{pmatrix}. \quad (7\text{-}8)$$

and

$$G = \begin{pmatrix} 0 & i\sigma_3 \\ -i\sigma_3 & 0 \end{pmatrix}. \quad (7\text{-}9)$$

$H_1 = \sum_n \delta t_n \boldsymbol{\sigma} \cdot \mathbf{T}_{K,n}$ gives rise to the general expression of the strain-induced gauge field:

$$\mathbf{A} = \frac{1}{v_0}\sum_n \delta t_n \mathbf{T}_{K,n}. \quad (7\text{-}10)$$

Note 8. Background SHG

During the SHG measurement of the strained graphene sample, other sources of SHG may also contribute to the measured data and complicate our analysis. These possible sources of SHG include those from nanopillar substrate or from the underlying Al_2O_3 layer. Therefore, it is important to exclude these effects.

Supplementary Figure S6 shows the emission spectrum from bare nanopillars not covered by graphene using the same experimental conditions described in Supplementary Note 2. The measured SHG signal cannot be distinguished from the background. Similarly, the measured SHG from Al_2O_3 is also lower than noise level. This confirms that the substrate does not contribute to our measurement of SHG in strained graphene, and therefore the substrate induced SHG can be excluded.

Note 9. Estimation of conversion efficiency and second-order susceptibility

The conversion efficiency is estimated by finding the ratio between the SHG power $P_{2\omega}$ and the incident laser power $P_{\omega,\mathrm{I}}$ using the Andor detector and a power meter[9]. The detector allows us to measure the emission spectrum of SHG and the spectrum of the reflected laser directly. When measuring the spectrum of the reflected laser, multiple neutral density filters must be used and the integration time of the detector must be set at the minimum, so as not

to damage the detector. The reduction of the measured laser power due to the filters and the short integration time needs to be accounted for when calculating the conversion efficiency. Thereafter, the spectrum of the SHG and reflected laser allows us to get the ratio between $P_{2\omega}$ and the reflected laser power $P_{\omega,\mathrm{r}}$.

Next, the ratio of the incident laser power $P_{\omega,i}$ to the reflected laser power $P_{\omega,r}$ is measured directly using a power meter. The conversion efficiency can then be estimated using the formula: $\eta = \frac{P_{2\omega}}{P_{\omega,\mathrm{r}}} \times \frac{P_{\omega,r}}{P_{\omega,\mathrm{i}}}$.

The sheet second-order susceptibility $\chi_{\mathrm{sh}}^{(2)}$ of our strained graphene sample can be obtained immediately using Equation (6-1) once the conversion efficiency is known [10,11].

$$|\chi_{\mathrm{sh}}^{(2)}| = \sqrt{\frac{P_{2\omega} \times c \times \varepsilon_0 \times RR \times A \times \Delta\tau \times \lambda^2 \times (1+n)^6}{64\sqrt{2}\pi^2 \times P_{\omega}^2 \times S \times n^3}} \qquad (9\text{-}1)$$

where c is the speed of light in vacuum, ε_0 is the electric permittivity of vacuum, RR is the repetition rate of the pump laser, A is the pump spot area, $\Delta\tau$ is the pulse width, λ is the pump wavelength, n is the refractive index of graphene, S is the shape factor of Gaussian pulses.

Alternatively, the value of sheet second-order susceptibility of the strained graphene sample $\chi_{\mathrm{sh,Gr}}^{(2)}$ can also be estimated by benchmarking against that of hBN. Supplementary Figure S7 shows the SHG spectrum of strained graphene and hBN under the same measurement conditions. Pristine hBN on a flat SiO_2 substrate was chosen to estimate the second-order susceptibility of our strained graphene sample, since its susceptibility value is well documented[11,12]. During the measurement, the strongest response from the polarization-resolved measurement is taken for comparison. Both samples were measured at the same temperature of 4 K in vacuum condition. Further work may be done to compare the second-order susceptibility of our strained graphene with other non-centrosymmetric 2D materials, including 2D ferroelectrics. We can then use Equation (6-2) to estimate $\chi_{\mathrm{sh,Gr}}^{(2)}$ [13].

$$\chi^{(2)}_{\mathrm{sh,Gr}} = \frac{\sqrt{I_{\mathrm{Gr}}(2\omega)}}{\sqrt{I_{\mathrm{hBN}}(2\omega)}}\chi^{(2)}_{\mathrm{sh,hBN}} \qquad (9\text{-}2)$$

By taking the value of $\chi^{(2)}_{\mathrm{sh,hBN}}$ to be 1.4×10^{-20} $\mathrm{m^2V^{-1}}$ [11], we estimated that our strained graphene sample has an average value of $\chi^{(2)}_{sh,Gr}$ of 1.82×10^{-20} $\mathrm{m^2V^{-1}}$. Thereafter, we can easily calculate the monolayer second-order nonlinear susceptibility $\chi^{(2)}$ of strained graphene to be approximately 1.3×10^{-11} $\mathrm{mV^{-1}}$.

Note 10 Polarization-resolved SHG

Since SHG from strained graphene arises from the strain-induced sublattice polarization, its polarization pattern is expected to be different from those previously reported in pristine graphene. Pristine graphene on a substrate has C_{6v} symmetry[14], whereas graphene transferred on a step-edge of a substrate is expected to belong to the C_s symmetry group because the sublattice polarization distributed along the step-edge results in one mirror plane perpendicular to the edge.

To verify this hypothesis, we measure the polarization-resolved SHG on a piece of graphene that is transferred on a trench structure at a temperature of 4 K. Figure S8 shows the SEM image of graphene transferred on a SiO_2 trench with a depth of ~50 nm. At the two long parallel edges of the trench, graphene experiences a non-uniform out-of-plane strain that generates PMF and sublattice polarization. Figure S9 illustrates the optical setup of our polarization-resolved measurements. A half-wave plate is placed between the input laser and the cryostat to adjust the polarization of the input laser. An analyzer (linear polarizer) is placed between the cryostat and the detector. During the measurements, the planes of polarization of the incident laser and the detected SHG are rotated over 180˚ while constantly being kept in a parallel direction. The polar plot of the detected SHG intensity over 360˚ (Fig. S10) is then plotted by repeating the measured data twice, since the plane of polarization repeats itself every 180˚.

Our polarization-resolved measurements reveal that the SHG response is highly anisotropic, exhibiting a two-fold symmetry as a function of the sample's azimuthal angle ϕ, as shown in Fig. S10. Such a pattern of the polarized SHG is attributed to strain introduced by the trench.

To understand why graphene produces such a polarization-dependent SHG pattern, we start from the optical response in general, which can be described by the polarization $\mathbf{P}(t)$:

$$\mathbf{P}(\omega, 2\omega, \cdots) = \chi^{(1)} \cdot \mathbf{E}(\omega) + \chi^{(2)}: \mathbf{E}(\omega)\mathbf{E}(\omega) + \cdots, \tag{10-1}$$

where $\chi^{(2)}$ is a rank-3 tensor of the frequency-dependent nonlinear susceptibility. We now analyse the symmetry constraint of the susceptibility tensor element $\chi^{(2)}_{abc}$. In the electric-dipole approximation, the inversion symmetry breaking is essential to the non-zero elements $\chi^{(2)}_{abc}$. The polarization components (P_x, P_y, P_z) can be expressed as:

$$\begin{pmatrix} P_x \\ P_y \\ P_z \end{pmatrix} = \begin{pmatrix} \chi_{xxx} & \chi_{xyy} & \chi_{xzz} & \chi_{xxy} & \chi_{xxz} & \chi_{xyz} \\ \chi_{yxx} & \chi_{yyy} & \chi_{yzz} & \chi_{yxy} & \chi_{yxz} & \chi_{yyz} \\ \chi_{zxx} & \chi_{zyy} & \chi_{zzz} & \chi_{zxy} & \chi_{zxz} & \chi_{zyz} \end{pmatrix} \begin{pmatrix} E_x E_x \\ E_y E_y \\ E_z E_z \\ 2E_x E_y \\ 2E_x E_z \\ 2E_y E_z \end{pmatrix}. \tag{10-2}$$

Generally, we have 27 independent matrix elements in the susceptibility tensor $\chi^{(2)}$. With the constraint of the crystal symmetry, we can reduce the elements by combining the identical matrix elements. Considering a rotation angle ϕ of the sample, we denote $p_i = \chi_{ijk} e_j e_k$ as the polarization induced in the sample frame, and P_i as the polarization in the laboratory frame. We can write:

$$\begin{pmatrix} P_x \\ P_y \end{pmatrix} = \begin{pmatrix} \cos\phi & -sin\phi \\ \sin\phi & \cos\phi \end{pmatrix} \begin{pmatrix} p_x \\ p_y \end{pmatrix},$$

$$\begin{pmatrix} e_x \\ e_y \end{pmatrix} = \begin{pmatrix} \cos\phi & sin\phi \\ -\sin\phi & \cos\phi \end{pmatrix} \begin{pmatrix} E_x \\ E_y \end{pmatrix}. \tag{10-3}$$

By combining the above equations, we can easily obtain the following relationship:

$$\frac{P_{xx}}{E_x^2} = -(D + 2C)\cos^2\phi \sin\phi + (2F + G)\sin^2\phi \cos\phi + A\cos^3\phi$$
$$- B\sin^3\phi,$$
$$\frac{P_{xy}}{E_y^2} = (2C - B)\cos^2\phi \sin\phi - (2F - A)\sin^2\phi \cos\phi + G\cos^3\phi$$
$$- D\sin^3\phi, \tag{10-4}$$

where $A = \chi_{xxx}$, $B = \chi_{yyy}$, $C = \chi_{xxy} = \chi_{xyx}$, $D = \chi_{yxx}$, $F = \chi_{yyx} = \chi_{yxy}$, and $G = \chi_{xyy}$. Additionally, we note that SHG intensity $I_{SHG} \propto |P^2|$. In different crystal structure with different point group symmetry, the susceptibility elements will have further constraints. Graphene transferred on the trench is expected to belong to the C_s symmetry group. For this symmetry group, the only non-zero susceptibility elements are B, C and D, and the expected SHG response is shown in Fig. S10.

Note 11 SHG from graphene on pillar arrays with different heights

To observe the effect of PMF strength on the second-order response from graphene, we transfer graphene onto three nanopillar arrays with different pillar heights. The three pillar blocks have a pillar height of 120 nm, 210 nm, and 300 nm respectively (Fig. S11), and each pillar has the same length and width. The three pieces of graphene are all oriented in a way such that the zigzag and armchair orientations are aligned to adjacent edges of a nanopillar. Due to the difference in the pillar height, graphene layers in the three samples experience different PMF strength. To illustrate the relationship between pillar heights and PMF strength, we theoretically calculate the 2D distribution of PMF using the same method mentioned in the manuscript. Figure S12 shows the PMF distribution across the three nanopillars with a height of 120 nm, 210 nm, and 300 nm respectively. As expected, the tallest (300 nm) nanopillar array produces a maximum PMF of ~150 T in graphene, which is almost 4 times that of the shortest (120 nm) nanopillar.

We then perform the SHG measurements on the three samples to compare the SHG intensities from the three samples at the same pumping condition at room temperature. As shown in Fig. S13, we find out the ratio of their SHG intensities to be around 1∶ 1.35 ∶ 2.23.

To corroborate our experimental findings, we simulate the expected $\chi^{(2)}$ based on the 2D PMF distribution. Figure S14 shows the expected 2D distribution of $\chi^{(2)}$ based on the PMF distribution. We find out that a higher nanopillar produces a larger average value of $\chi^{(2)}$ at the edges. This can be explained with the fact that a large PMF forces the LDOS of pseudo-Landau levels to be sharper, and therefore enables a stronger resonance between the pseudo-Landau levels and produces a large SHG intensity.

Note 12 SHG from strained graphene under gating

We measured the SHG signals from both unstrained and strained graphene under gating. Figure S15 shows the SEM image of our sample. Monolayer graphene was transferred on a SiO_2 trench, before metal contacts were made to apply back gate to our sample. We then separately measure and compare the emission spectrum of graphene at the edges of the trench and at the flat area at room temperature. Figure S16a and b shows the emission spectrum for unstrained graphene and strained graphene respectively. For both samples, the broadband emission spanning across the entire spectrum comes from the ultrafast emission of the thermalized carriers[15]. For graphene at the edges of the trench, sharp peaks of SHG can be observed. The SHG emission intensity significantly decreases when the back gate voltage V_{bg} is tuned away from the Dirac point voltage V_{Dirac}, while the ultrafast broadband emission is slightly decreased. The suppression of the emission can be explained by the effect of Pauli blocking[16]. The reduction of SHG intensity is much more significant than ultrafast thermal emission, probably because the SHG process relies heavily on the strong resonant transitions involving pseudo-Landau levels. Tuning V_{bg} away from V_{Dirac} may suppress resonant optical transition channels and drastically reduces the SHG intensity. We think that back gate tuning of SHG in pseudo-Landau quantized graphene is an interesting research topic worth further study.

Supplementary Figures

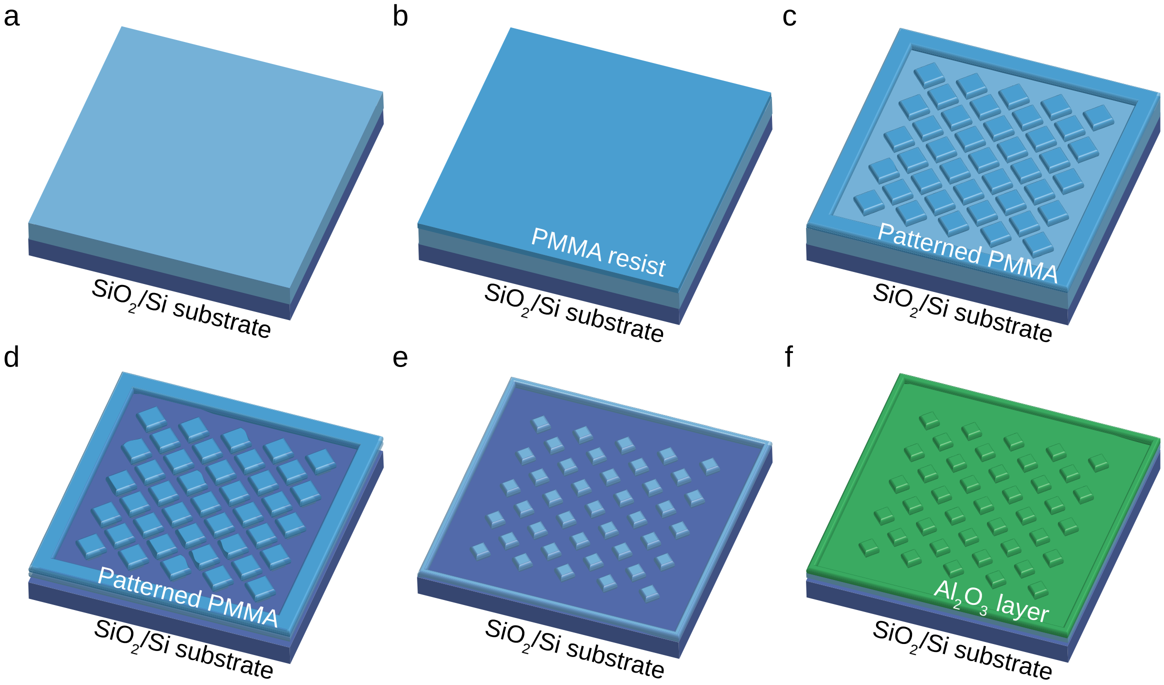

Supplementary Figure S1 | Fabrication process of nanostructured substrate. a-b, The PMMA resist was covered on a 300-nm-thick SiO_2/Si substrate. **c,** Patterned PMMA is used as an etch mask. **d,** The sample was then soaked in BOE for 3 min 30 sec to etch the SiO_2. **e,** The PMMA layer was removed using acetone, IPA, DI water, and O_2 plasma. **f,** a layer of 20-nm thick Al_2O_3 was deposited using ALD.

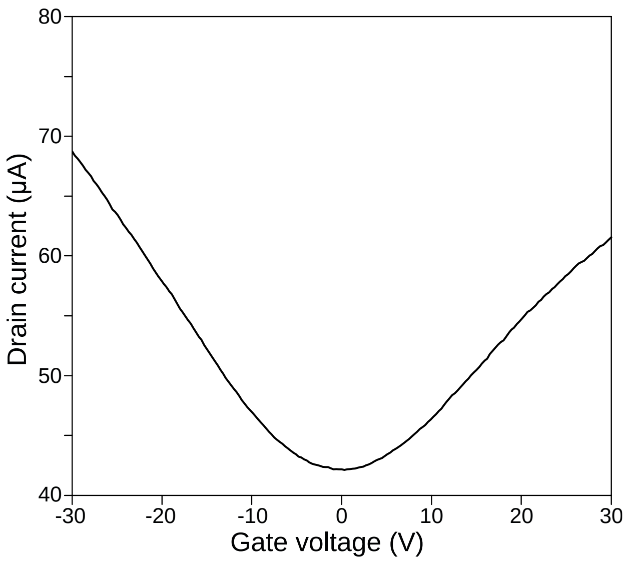

Supplementary Figure S2 | I-V characteristics of graphene on Al_2O_3. The I_d-V_g characteristics of a graphene device on Al_2O_3 shows that Dirac point is close to 0, which indicates that the underlying Al_2O_3 does not introduce discernible charges in graphene. Therefore, substrate-induced effects can be excluded from subsequent experiments.

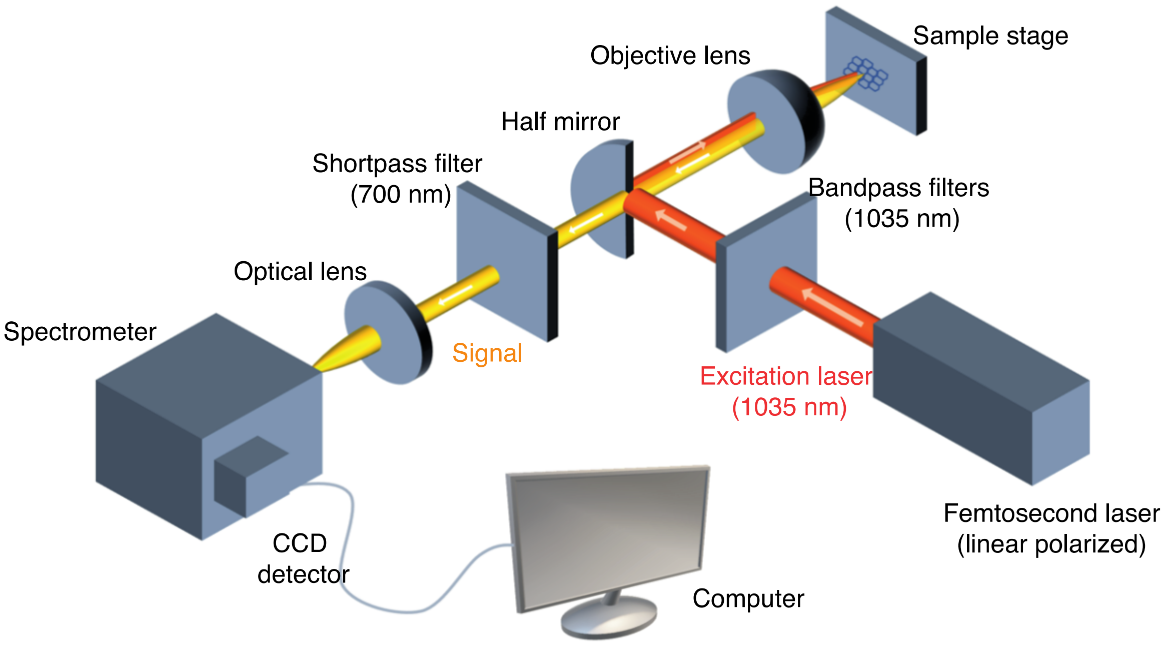

Supplementary Figure S3 | Measurement setup. The schematic illustration of the measurement setup for temperature-dependent second harmonic generation.

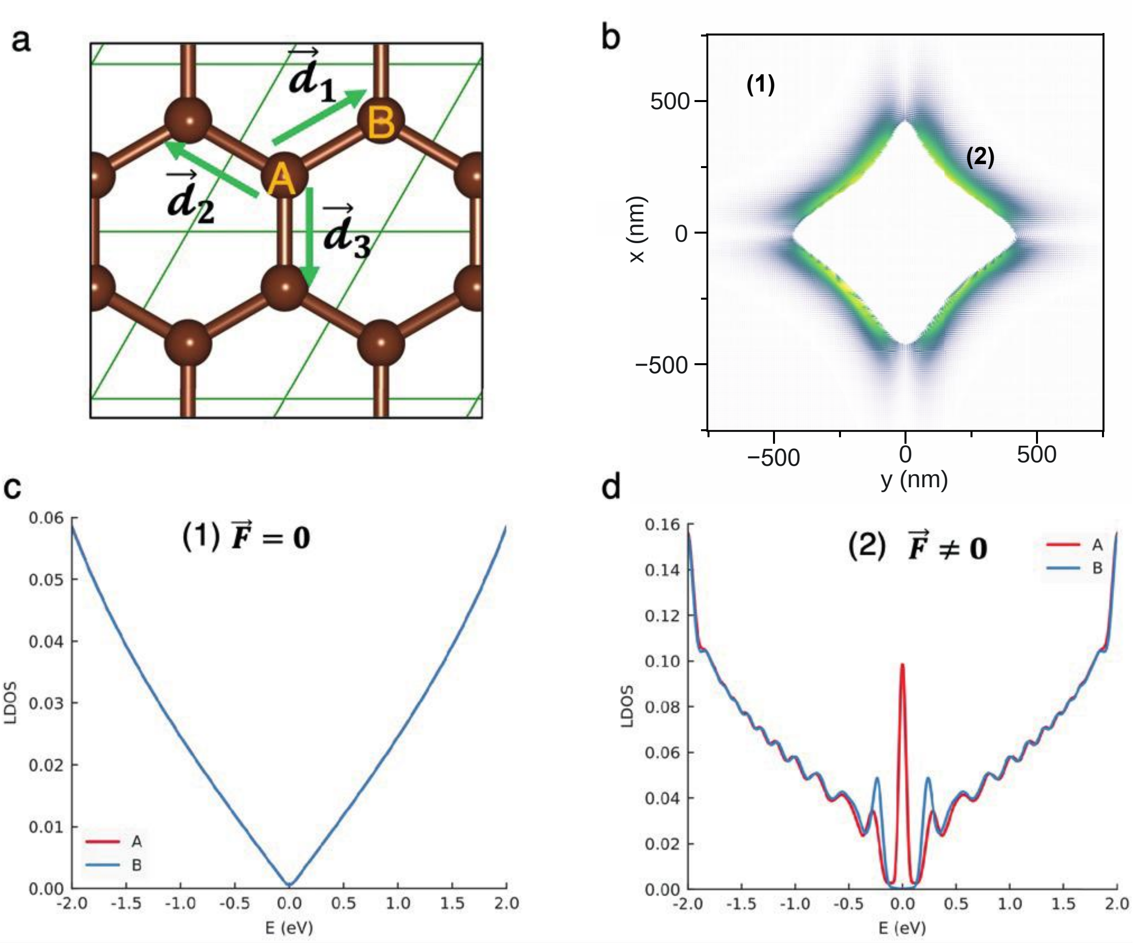

Supplementary Figure S4 | Schematic illustration of lattice polarization. a, $\vec{\delta}_1$, $\vec{\delta}_2$, and $\vec{\delta}_3$ are defined as the unit vectors along the three armchair directions, which are equally separated by 120°. **b,** 2D mapping of sublattice polarization in graphene on a single nanopillar structure. The polarization is almost 0 in areas away from the nanopillar (1) and is non-zero at the edges of the nanopillar (2). **c and d,** Distribution of local density of states in area (1) and (2), respectively.

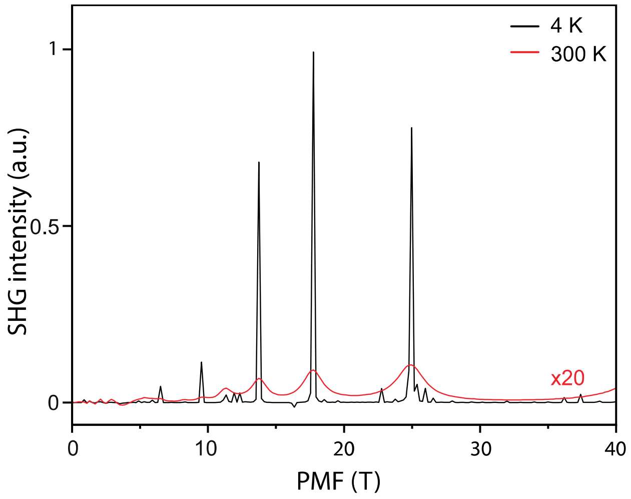

Supplementary Figure S5 | Second-order response at different strengths of pseudo-magnetic fields. The second-order response changes drastically when the temperature and the strength of pseudo-magnetic fields changes. Resonant second harmonic generation only occurs at specific magnitudes of pseudo-magnetic field strengths. When the temperature is reduced, the resonance of second harmonic generation becomes drastically stronger.

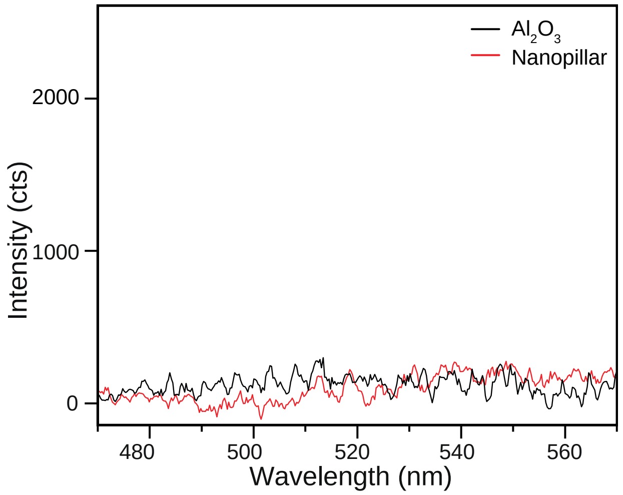

Supplementary Figure S6 | Negligible second order generation from substrate. The measured second order generation from both the bare nanopillar array and bare oxide surface is almost 0 (below noise level), thereby confirming that the second order generation is from strained graphene.

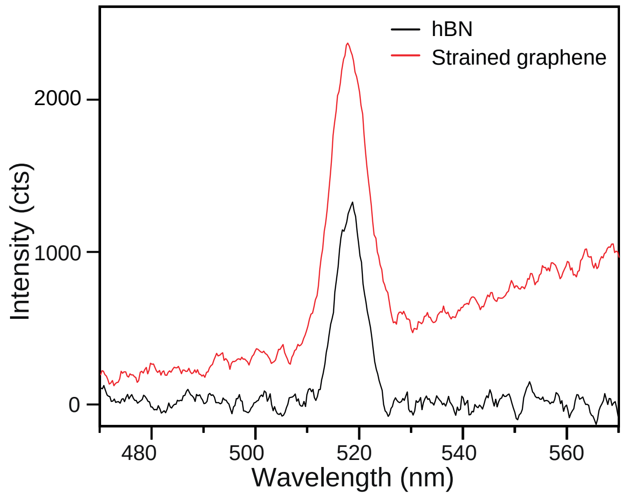

Supplementary Figure S7 | Comparison of second order generation from strained graphene and hexagonal boron nitride. The second order generation measured from strained graphene is even higher than that of hexagonal boron nitride. The value of second-order nonlinear susceptibility of strained graphene can be estimated by referencing that of hexagonal boron nitride.

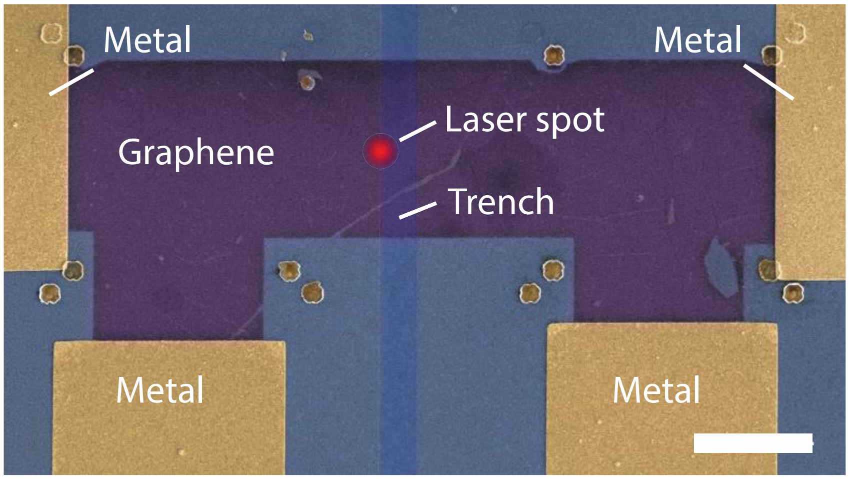

Figure S8 | SEM image of graphene transferred on a SiO_2 trench. SiO_2 trench has a depth of ~50 nm. Scale bar: 20 μm.

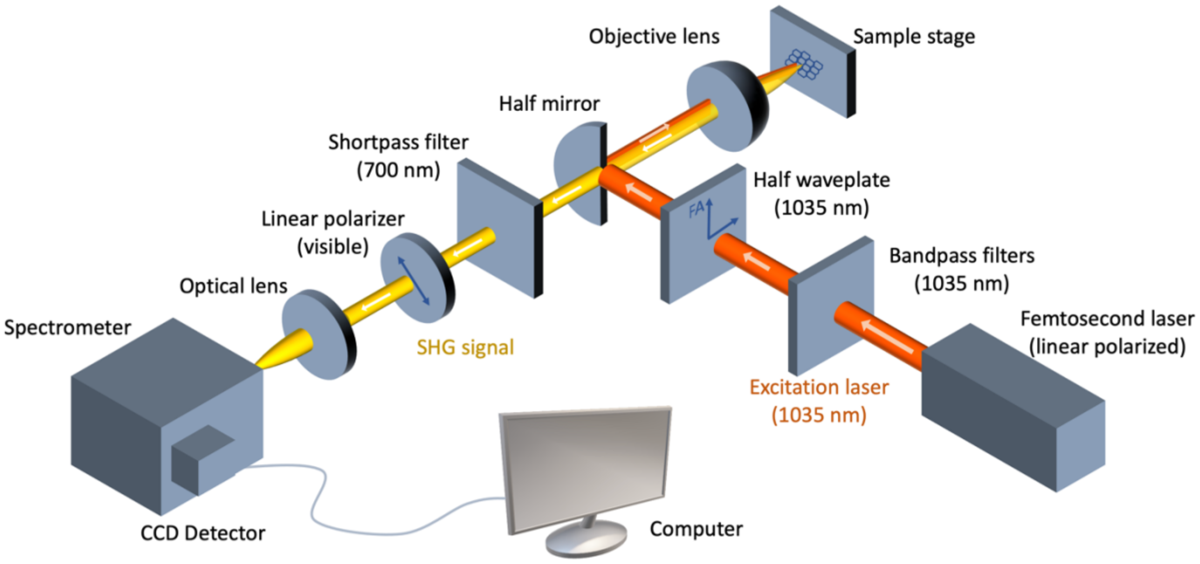

Figure S9 | Experimental setup of polarization-resolved SHG measurements.

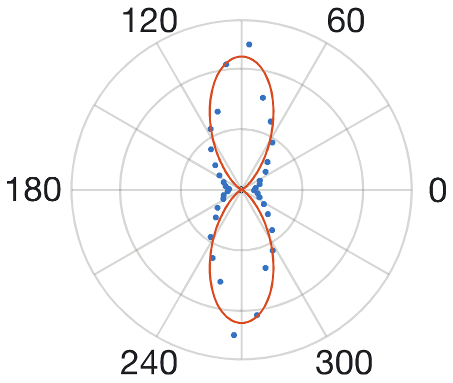

Figure S10 | Polar plot of SHG intensity from graphene on trench structure as a function of the azimuthal angle. The angle $\phi = 0°$ corresponds to the orientation where the polarization of the pump laser lies along the long edges of the trench. The dots are experimental data and the curve is the fit based on symmetry analysis.

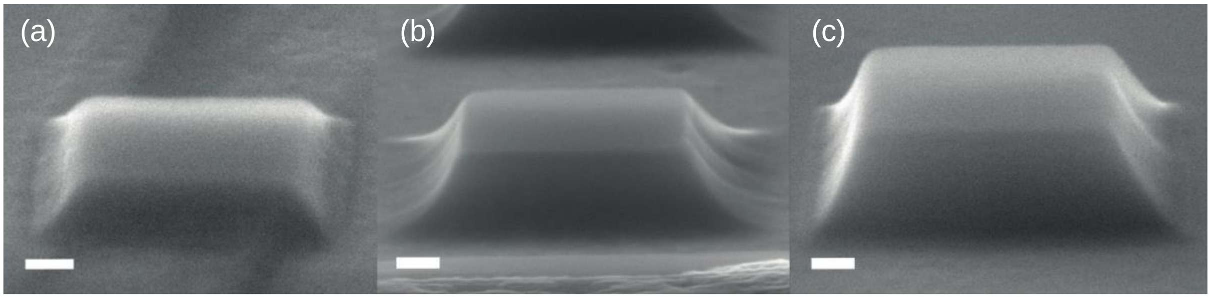

Figure S11 | SEM images of SiO_2 pillars for PMF-dependent SHG studies. The three pillar arrays have an average height of (a) 120 nm (b) 210 nm (c) 300 nm, respectively. Scale bar: 150 nm.

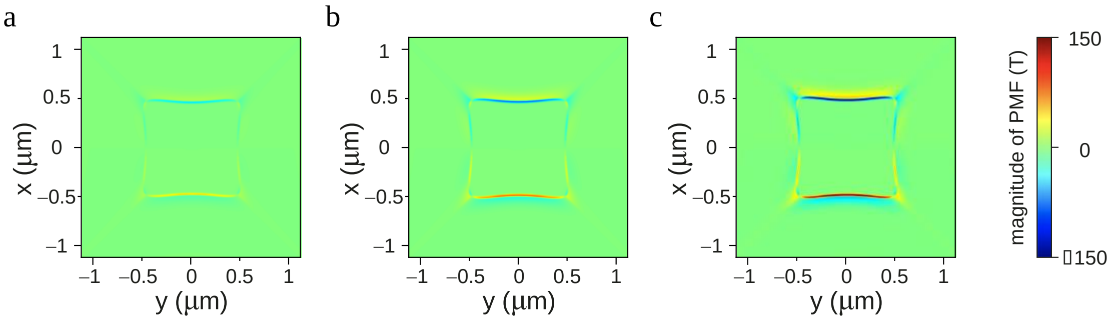

Figure S12 | 2D mapping of PMF. PMF in graphene transferred on pillar arrays with a height of (a) 120 nm, (b) 210 nm, and (c) 300 nm.

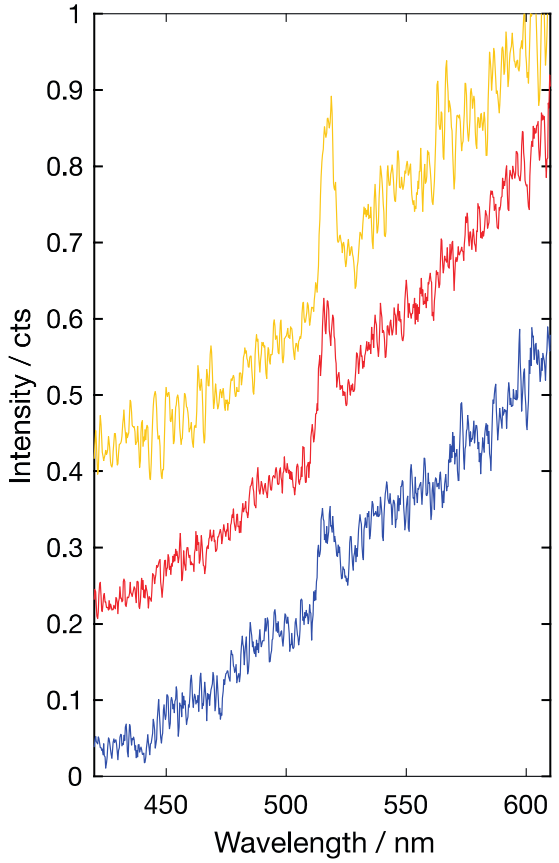

Figure S13 | SHG spectrum. Emission spectra of graphene on pillar arrays with an average height of 120 nm (blue), 210 nm (red) and 300 nm (yellow).

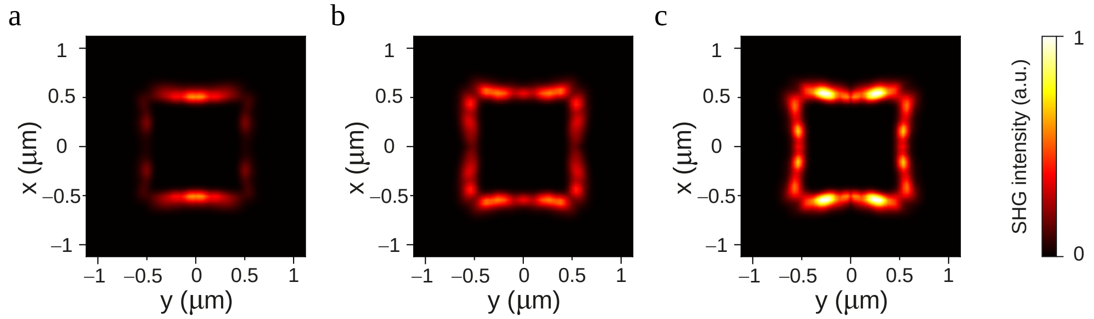

Figure S14 | 2D mapping of second-order susceptibility. Theoretical simulation of second-order susceptibility in a single reconstructed pillar with a height of (a) 120 nm, (b) 210 nm, and (c) 300 nm.

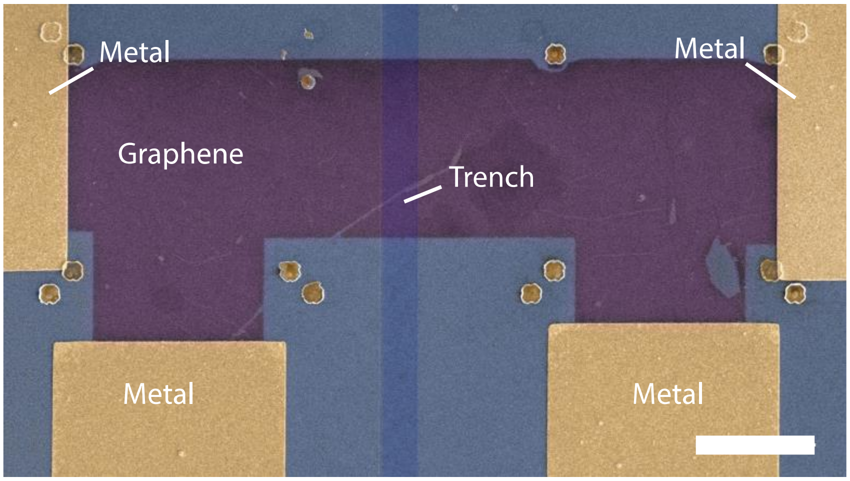

Figure S15 | SEM image of graphene transferred on a SiO_2 trench. SiO_2 trench has a depth of ~50 nm. Scale bar: 20 μm.

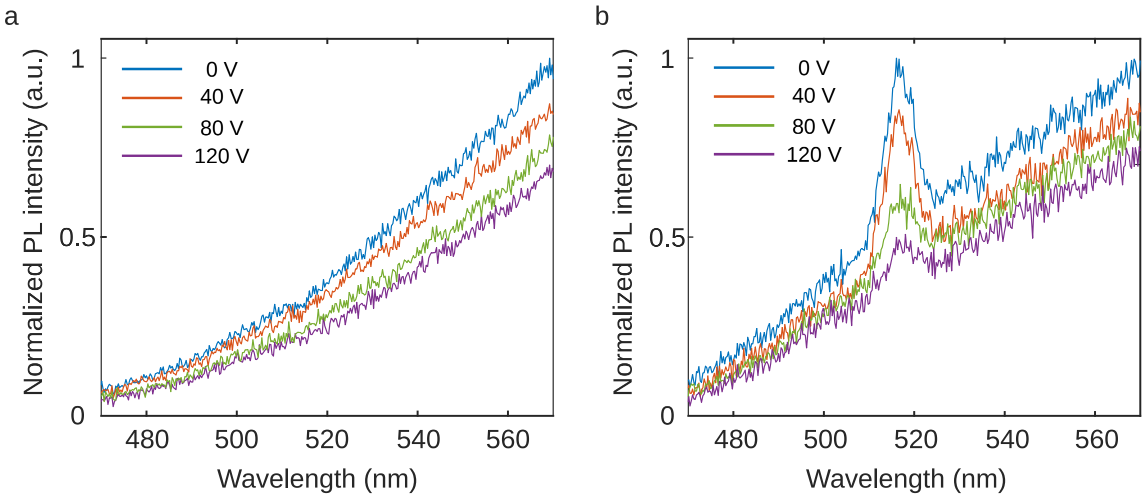

Figure S16 | Emission spectrum with back gate tuning. Photoluminescence spectrum of (a) unstrained graphene (b) strained graphene under gating from V_{Dirac} to V_{Dirac} +120 V.

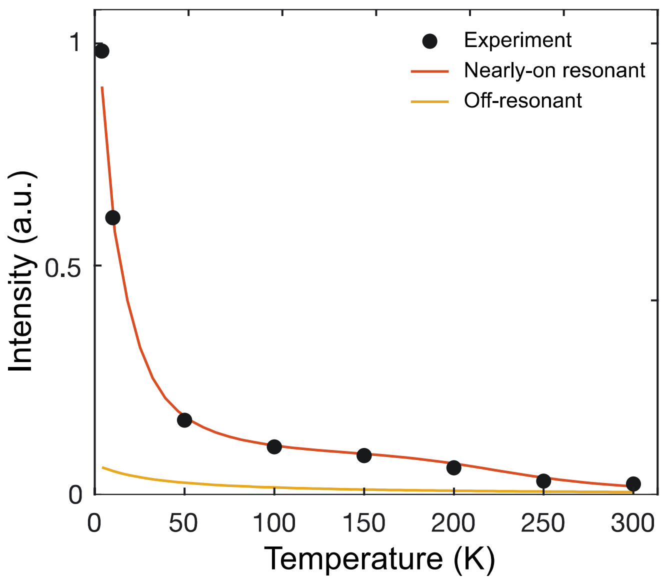

Supplementary Figure S17 | Plot of SHG intensity as a function of temperature. Black circles: experimental data; Red curve: calculated data considering all possible inter-pLL resonant SHG at all locations across the nanopillar; Yellow curve: calculated data without considering resonances.

References

1. de Juan, F., Sturla, M. & Vozmediano, M. A. H. Space dependent Fermi velocity in strained graphene. *Phys Rev Lett* **108**, 227205 (2012).
2. Leitsmann, R., Schmidt, W. G., Hahn, P. H. & Bechstedt, F. Second-harmonic polarizability including electron-hole attraction from band-structure theory. *Phys Rev B Condens Matter Mater Phys* **71**, 195209 (2005).
3. Xu, H., Wang, H., Zhou, J. & Li, J. Pure spin photocurrent in non-centrosymmetric crystals: bulk spin photovoltaic effect. *Nat Commun* **12**, 4330 (2021).
4. Vandelli, M., Katsnelson, M. I. & Stepanov, E. A. Resonant optical second harmonic generation in graphene-based heterostructures. *Phys Rev B* **99**, 165432 (2019).
5. Wendler, F., Knorr, A. & Malic, E. Ultrafast carrier dynamics in Landau-quantized graphene. *Nanophotonics* **4**, 224–249 (2015).
6. Lu, T. *et al.* Temperature-dependent photoluminescence in light-emitting diodes. *Sci Rep* **4**, 6131(2014).
7. Wendler, F. & Malic, E. Carrier-phonon scattering in Landau-quantized graphene. *Phys Status Solidi B Basic Res* **251**, 2541–2544 (2014).
8. Wendler, F., Knorr, A. & Malic, E. Resonant carrier-phonon scattering in graphene under Landau quantization. *Appl Phys Lett* **103**, 253117 (2013).
9. Shree, S. *et al.* Interlayer exciton mediated second harmonic generation in bilayer MoS_2. *Nat Commun* **12**, 6894 (2021).
10. Woodward, R. I. *et al.* Characterization of the second- and third-order nonlinear optical susceptibilities of monolayer MoS2 using multiphoton microscopy. *2d Mater* **4**, 011006 (2017).
11. Kim, S. *et al.* Second-harmonic generation in multilayer hexagonal boron nitride flakes. *Opt Lett* **44**, 5792 (2019).
12. Li, Y. *et al.* Probing symmetry properties of few-layer MoS2 and h-BN by optical second-harmonic generation. *Nano Lett* **13**, 3329–3333 (2013).
13. Cunha, R. *et al.* Second harmonic generation in defective hexagonal boron nitride. *Journal of Physics Condensed Matter* **32**, 19LT01 (2020).
14. Dean, J. J. & van Driel, H. M. Second harmonic generation from graphene and graphitic films. *Appl Phys Lett* **95**, 261910 (2009).
15. Lui, C. H., Mak, K. F., Shan, J. & Heinz, T. F. Ultrafast photoluminescence from graphene. *Phys Rev Lett* **105**, 127404 (2010).
16. Huang, D. *et al.* Gate switching of ultrafast photoluminescence in graphene. *Nano Lett* **18**, 7985–7990 (2018).
